# Genetic diversity and complexity of *Plasmodium falciparum* infections in the microenvironment among siblings of the same household in North-Central Nigeria

**DOI:** 10.1186/s12936-020-03415-1

**Published:** 2020-09-16

**Authors:** Segun Isaac Oyedeji, Peter Usman Bassi, Samuel Adeola Oyedeji, Olusola Ojurongbe, Henrietta Oluwatoyin Awobode

**Affiliations:** 1 Molecular Genetics and Parasitology Unit, Department of Animal & Environmental Biology, Federal University Oye-Ekiti, Oye-Ekiti, Nigeria; 2 Department of Pharmacology and Therapeutics, University of Abuja, Abuja, Nigeria; 3 Department of Zoology, University of Ilorin, Ilorin, Nigeria; 4 Department of Medical Microbiology and Parasitology, Ladoke Akintola University of Technology, Osogbo, Nigeria; 5 Parasitology Unit, Department of Zoology, University of Ibadan, Ibadan, Nigeria

**Keywords:** *Plasmodium falciparum*, Genetic diversity, Malaria, Multiplicity of infection, *MSP*-2, Transmission intensity, Micro-environment, Nigeria

## Abstract

**Background:**

*Plasmodium falciparum* parasites are known to exhibit extensive genetic diversity in areas of high transmission intensity and infected individuals in such communities often harbour several complex mixtures of parasite clones with different genetic characteristics. However, in the micro-environment, the extent of genetic diversity of *P. falciparum* parasites remain largely unknown. In this study therefore, the complexity of *P. falciparum* infections in households was investigated among symptomatic siblings, living under the same roof in north-central Nigeria.

**Methods:**

Children were enrolled into the study if they were at least two from a household and presented with symptoms of uncomplicated malaria. Clinical malaria was confirmed by light microscopy of Giemsa-stained thick and thin blood films. Genomic DNA was isolated from blood spots on filter paper. Molecular characterization of *P. falciparum* isolates was done by allele-specific nested PCR of the highly polymorphic merozoite surface protein-2 (*msp-2*) gene.

**Results:**

Ninety-three children from 43 households were enrolled into this study. A total of 26 different *msp-2* alleles were identified from 215 fragments (range: 180–480 bp). Majority of the isolates [65.6% (n = 61)] were polyclonal infections consisting of 2–6 clones and were significantly more common with the FC27 allelic family (*p* = 0.036). The multiplicity of infection (MOI) per household ranged from 1.0 to 4.5 while the overall MOI in the study population was 2.31. The pattern of distribution of *msp-2* allele types among the households fell into two categories: households where both *msp-2* allele types (FC27 and 3D7) were present; households where only one *msp-2* allele type (FC27 or 3D7) was present. Majority of the households [88.4% (n = 38)], had both *msp-2* allele types but they were disproportionately distributed among the children while in a few households [11.6% (n = 5)], all the children were infected with only one type of *msp-2* allele.

**Conclusion:**

These findings showed that *P. falciparum* isolates exhibit remarkable degree of genetic diversity in the micro-environment and are composed mainly of multiclonal infections, which is an indication of a high ongoing parasite transmission. This suggests that the micro-environment is an important area of focus for malaria control interventions and for evaluating intervention programmes.

## Background

Malaria is a major parasitic disease of public health concern in tropical and sub-tropical regions of the world. Human malaria is caused by *Plasmodium falciparum*, *Plasmodium vivax*, *Plasmodium ovale*, *Plasmodium malariae*, and *Plasmodium knowlesi* [1, 2]. Of these, *P*. *falciparum* occurs predominantly in Africa and is responsible for most of the severe and life-threatening forms of the disease [3, 4]. The ability of *P. falciparum* to develop resistance to almost all available anti-malarials, the emergence of insecticide resistant mosquitoes, and the non-availability of a malaria vaccine have been major obstacles to the effective control and eradication of malaria. Considerable progress was made in the control of malaria between 2000 and 2015 when global malaria mortality and incidence rates fell by 62 and 41%, respectively [5]. The massive rollout of mosquito nets coupled with anti-malarial drugs and the use of indoor residual spraying of insecticides was attributed to the recorded success. Despite commendable gains, which unfortunately were not sustained, malaria still affects hundreds of millions of people globally [6, 7]. About 228 million cases were estimated to have occurred worldwide in 2018, resulting in the death of over 400,000 individuals, most of which were in sub-Saharan Africa [8]. Nigeria, the most populous nation in sub-Saharan Africa, is responsible for the highest burden worldwide: deaths of approximately 30% of children aged under 5 years, 25% infant mortality, and 11% maternal death. A major tool used by *P. falciparum* parasites to undermine control measures such as chemotherapy, insecticide-treated nets, and the development of an effective vaccine is its characteristic phenotypic and genetic diversity.

*Plasmodium falciparum* exhibits enormous genetic diversity in natural populations, and this is evident in the number of antigenically diverse parasite populations found among infected individuals in malaria-endemic regions. Genetic diversity in the parasite population is characteristically a reflection of the transmission intensity in an area and is required for the acquisition of protective immunity against malaria [9–12]. Individuals living in areas of high or intense malaria transmission are frequently infected with several complex mixtures of distinct parasite clones [13–17]. On the other hand, the majority of infections in low transmission areas are monoclonal [18–20].

Genetic diversity in natural populations of the malaria parasites has been characterized in several studies using polymorphic, unlinked genetic markers in the parasite genome. Notable amongst these markers are the genes encoding surface antigens found at the developmental stages of the parasite life cycle, particularly the blood-stage antigens merozoite surface protein 1 (MSP-1) and (MSP-2), which are known to be highly polymorphic. Several studies have utilized PCR typing of the *msp-2* locus alone or together with the *msp-1* locus to assess genetic diversity and complexity of *P. falciparum* infections in different communities with varying transmission intensities and among infected individuals with different clinical presentations of malaria [21–29]. However, little is known about the diversity of *P. falciparum* infections at the level of the micro-environment, among members of the same household. The aim of this study, therefore, was to determine the genetic diversity of *P. falciparum* isolates in children of the same household, living under the same roof, in Lafia, north-central Nigeria, using the highly polymorphic *msp-2* gene.

## Methods

### Study area and population

This study was conducted in Lafia, a city located within the middle belt region in north-central Nigeria. Lafia lies within Nigeria’s Guinea savannah ecological zone and in this area malaria is known to be endemic and perennial as described previously [30, 31]. The main vectors serving as agents of malaria transmission in this region are *Anopheles gambiae *sensu stricto (*s.s*.), *Anopheles arabiensis*, *Anopheles funestus*, *Anopheles moucheti, Anopheles melas,* and *Anopheles nili* [2, 32]

Children aged 9 months to 12 years who presented with symptoms compatible with uncomplicated or mild malaria at the Dalhatu Araf Specialist Hospital Lafia, between 2006 and 2011, were enrolled into the study on "determinants of disease outcome in *P. falciparum*-infected children" after satisfying the inclusion criteria as follows:(i) at least 2 children of the same household living under the same roof; and, (ii) they must have been residing in the same house for at least 6 months. Uncomplicated or mild malaria was defined as symptomatic malaria with presentations that included chills, pyrexia at presentation (axillary temperature > 37.5 °C) or history of fever within the preceding 48 h, presence of asexual forms of *P. falciparum* in peripheral blood smears and absence of any indication of severe illness or vital organ dysfunction.

Participation in the study was voluntary. Before being included in the study, the study protocol was explained to the parent/guardian of the children from each family, and then informed consent was obtained. Ethical approval was obtained from the Ethics Committees of the Nasarawa State Ministry of Health and the Dalhatu Araf Specialist Hospital, Lafia, Nasarawa State, Nigeria, with reference numbers S/MH/519/VOL.1/84 and DASH/ADM/MR/VOL.1/0001, respectively. There were no selection criteria in the enrolment process of children in the households as children were registered at random. The first child presented by a parent during the enrolment process, irrespective of age or sex, was documented as child 1 for that particular household; the second child presented was child 2 and so on. All malaria-confirmed cases were appropriately treated by the Hospital clinicians.

### Sample collection and microscopy

Approximately 0.5 ml of venous blood samples were collected from each child for parasitological and haematological analyses. All samples were de-identified at the point of collection for the confidentiality of participants and only labelled with alphanumeric codes. Three drops of blood collected were blotted on labelled 3MM Whatman filter paper, air dried, individually sealed in plastic bags, and stored at room temperature until they were used for DNA extraction. Thick and thin blood smears were also prepared for microscopic examination. The slides were labelled and allowed to dry, after which the thin smears were fixed with methanol and subsequently allowed to dry. Slides were stained with freshly prepared 5% Giemsa stain for 20 min at room temperature [33] and examined under the microscope. Parasitaemia was quantified relative to 250 white blood cells (WBC) on thick films and estimated as parasites per µl assuming a mean WBC of 8000 per µl of blood. Smears were labelled negative if no parasites were seen after examination of 200 high-power field (HPF) at 1000 × magnification on a thick blood film. Blood haemoglobin levels were estimated by haematocrit measurement of packed cell volume (PCV) using the micro-haematocrit centrifuge.

### Parasite DNA extraction and genotyping of *Plasmodium falciparum msp-2* gene

DNA was isolated from the dried blood spots on filter paper using the QIAamp® DNA Mini Kit (Qiagen, Hilden, Germany) following the manufacturer’s instructions; 150 μl of distilled water was used to elute DNA, which was then stored at − 20 °C until use.

All samples were genotyped for *P. falciparum msp-2* gene by nested PCR according to previously described amplification procedures [31]. Briefly, a primary reaction and a second reaction (nested) was carried out on each sample. The primary reaction amplifies the entire coding region of the *msp-2* gene using the MSP2-1 and MSA2-4 primer pairs. Two sets of nested reactions were subsequently employed to amplify the central polymorphic region of the gene using the allele-specific primers FC 27-1/FC 27-2 and 3D7-1/3D7-2 for the FC27 and 3D7 allele types, respectively (Table 1). A third nested reaction was carried out with MSP2-2 and MSP2-3 primers to assess the frequency of isolates, which may be positive for *msp-2*, but not specific for FC27 or 3D7 allele type due to the polymorphic nature of the central region. The primary PCR mixture consisted of a final volume of 25 µl that included 12.5 µl of Go Taq® Green Master Mix (Promega Madison, USA), 2.0 µl of each primer (10 µM) and 5 µl of genomic DNA. The reaction was performed using the following cycling condition: initial denaturation step at 94 °C for 5 min followed by 35 cycles of 10 s at 94 °C, 30 s at 57 °C and 40 s at 72 °C and a final extension step of 72 °C for 3 min. All nested reactions were performed in a final volume of 25 µl containing 2.0 µl of PCR product from the primary reaction, 12.5 µl of Go Taq® Green Master Mix (Promega Madison, USA) and 2.0 µl of each primer (10 µM). The PCR cycling condition was: initial denaturation step at 94 °C for 5 s followed by 30 cycles of 10 s at 94 °C, 30 s at 57 °C and 40 s at 72 °C, and a final extension step of 72 °C for 3 min. Primer sequences were synthesized by Invitrogen Life Technologies, UK. All PCR assays were performed using a BIOMETRA TB1 ThermalCycler (Biotron, Göttingen Germany).

**Table 1:** The sequence of oligonucleotide primers used in this study

Primer name	Nucleotide sequence
Primary PCR	
MSP 2-1	5′-ATG AAG GTA ATT AAA ACA TTG TCT ATT ATA-3'
MSP 2-4	5′-TTA TAT GAA TAT GGC AAA AGA TAA AAC AAG-3'
Nested PCR	
MSP 2-2	5′-ACA TTC ATA AAC AAT GCT TAT AAT ATG AGT-3'
MSP 2-3	5′- GAT TAT TTC TAG AAC CAT GCA TAT GTC CAT -3'
FC27-1	5′-GCA AAT GAA GGT TCT AAT ACT AAT AG-3'
FC27-2	5′-GCT TTG GGT CCT TCT TCA GTT GAT TC-3'
3D7-1	5′-GCA GAA AGT AAG CCT TCT ACT GGT GCT-3'
3D7-2	5′-GAT TTG TTT CGG CAT TAT TAT GA-3'

PCR products were subjected to electrophoresis on 2% agarose and visualized under ultraviolet trans-illumination after staining with SYBR® Green. Fragment sizes were visually calculated relative to a standard size (100 bp) molecular weight DNA marker (New England Biolabs GmbH, Frankfurt am Main, Germany). The DNA fragments were grouped into bins if their fragment sizes were within 20 bp intervals.

### Statistical analysis

Data were analysed using XLSTAT Version 2019.1.2 [34]. The Student’s *t*-test was used to compare different normally distributed continuous variables. Numerical data not conforming to normal distribution were log-transformed. The multiplicity of infection (MOI) was defined as the minimum number of parasite genotypes per infected individual and was calculated as the mean number of PCR fragments or parasite genotypes per infected child. Clonality of infection was defined as the number of distinct PCR fragments per infected child. An infection was defined as polyclonal if more than one distinct PCR fragments or parasite genotypes were present in an isolate. Statistical significance was defined as *p* < 0.05.

## Results

### Baseline characteristics of study participants

A total of 93 children from 43 unrelated households/families were enrolled in this study. Each of the households had either 2 or 3 children with microscopically confirmed *P. falciparum* infection, although the majority (90.7%, 39/43) were those with 2 infected children. The clinical and demographic characteristics of the study participants are shown in Table 2. Of the enrolled 93 children, 59.1% (55/93) were males, while 40.9% (38/93) were females. Their age range was between 9 months and 12 years, while the mean age was 4.8 years. The mean PCV was 28% (ranged 18 to 42%), while the mean axillary temperature was 37.8 °C. The asexual parasitaemia ranged from 944 to 194,265 parasites per µl with a geometric mean parasite density of 21,418 per µl. There was a significant influence of the parasite density on axillary temperature (*p* = 0.041).

**Table 2:** Demographic and clinical characteristics of study participants (n = 93)

Characteristic	Value
Mean age (years)	6.8 (± 4.8)*
Gender ratio (male/female)	1.45 (55/38)
Mean axillary temperature (^o^C)	37.8 (± 0.84)*
Mean haematocrit (%)	28 (± 6.8)*
Geometric mean parasite density (μl)	21,418 (95% CI: 16,000–27,500)

* ± Standard deviation in parentheses

*CI* Confidence Interval

### Genetic diversity and complexity of *Plasmodium falciparum* infection in the study population

**Fig. 1:**
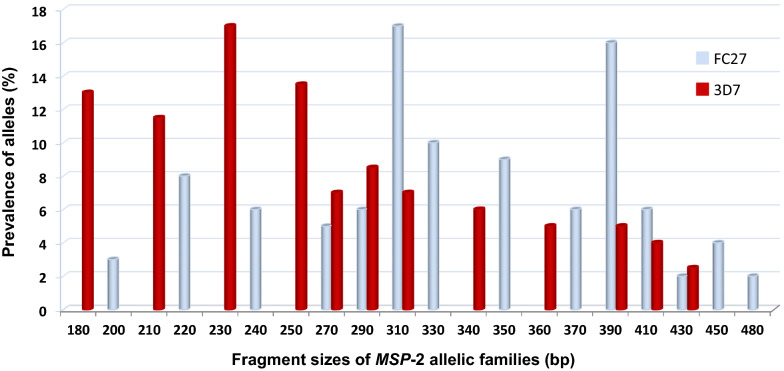
Distribution of *MSP*-2 alleles in isolates from the study participants

Isolates from all the children enrolled in the study were genotyped for allelic polymorphisms at the *MSP-*2 locus with 100% efficiency in the amplification reaction. The distribution of *MSP-*2 alleles among the 93 children from the 43 households studied showed high genetic diversity of the *P. falciparum* isolates as reflected in the inter-allelic as well as intra-allelic diversity. A total of 215 *MSP-*2 fragments were detected. The FC27 allele type was observed to be predominantly higher (60.5%, n = 130) than the 3D7 allele type (39.5%, n = 85). A total of 26 different *MSP-*2 alleles were identified from the 215 fragments after the fragments were grouped into bins within 20 bp intervals. The FC27 alleles included 14 different band sizes (range: 200–480 bp) of which the 310 bp was the most frequent (17.42%); while the 3D7 alleles included 12 different band sizes (range: 180–430 bp) and the most frequent (17.44%) was the 230 bp (Fig. 1). In terms of gender relationship, there was no significant difference in the distribution of FC27 and 3D7 alleles between males and females (*p* = 0.427).

**Fig. 2:**
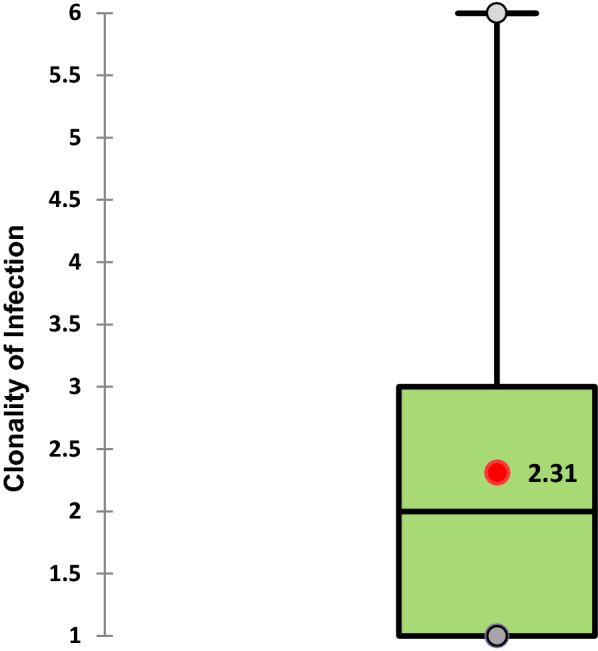
Box plot showing clonality of infection (clones per isolate) and the multiplicity of infection (MOI). The red dot indicates the multiplicity of infection (mean) while the middle line in the rectangular box shows the median value. The grey dots at the two ends show the maximum and the minimum values

The clonality of infection (number of distinct parasite clones) ranged from 1–6. The majority of the isolates (65.6%, 61/93) were polyclonal infections consisting of 2–6 clones; and were significantly more common with the FC27 allelic family (*p* = 0.036). The MOI was 2.31 (Fig. 2). No significant positive correlation was found between the clonality of infection/MOI and parasite density (Spearman's rank correlation coefficient = 0.06; *p* = 0.268).

### Genetic diversity and complexity of *Plasmodium falciparum* infection within households

**Fig. 3:**
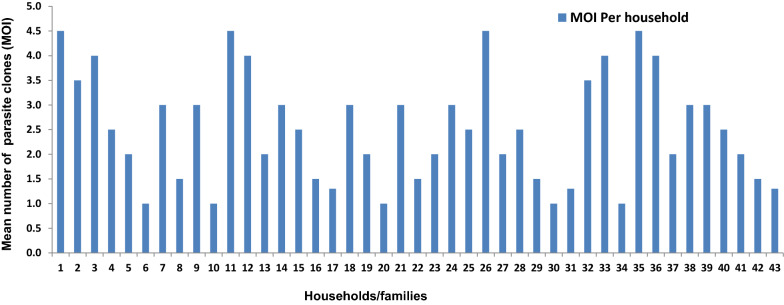
Multiplicity of infection (MOI) per household

The distribution of *msp-2* allelic families within the different households showed that *P. falciparum* infection at the micro-environment level is also genetically diverse. The total number of *msp-2* fragments per household ranged from 3 to 7, while the MOI per household ranged from 1.0 to 4.5 (Fig. 3). The majority of the households have a preponderance of the FC27 allele type. The pattern of distribution of the *msp-2* allelic families among the households could be categorized into two: households where both *msp-2* allele types (FC27 and 3D7) were present; households where only one *msp-2* allele type (FC27 or 3D7) was present.

**Fig. 4:**
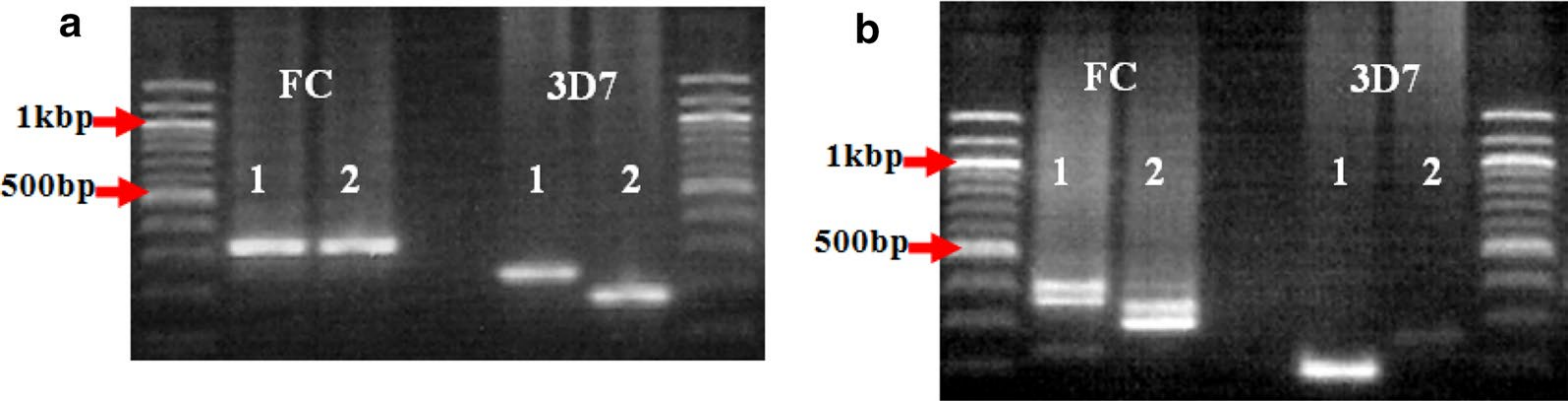
Alleles of the *MSP-2* gene showing both FC27 and 3D7 families in all children of same households. **a** Equal distribution of single clones of both FC27 and 3D7 alleles among participants in the same household. **b** Distribution of double clones of FC27 alleles and single clones of 3D7 alleles among participants in the same household. The numeric codes represent the children in the respective household

**Fig. 5:**
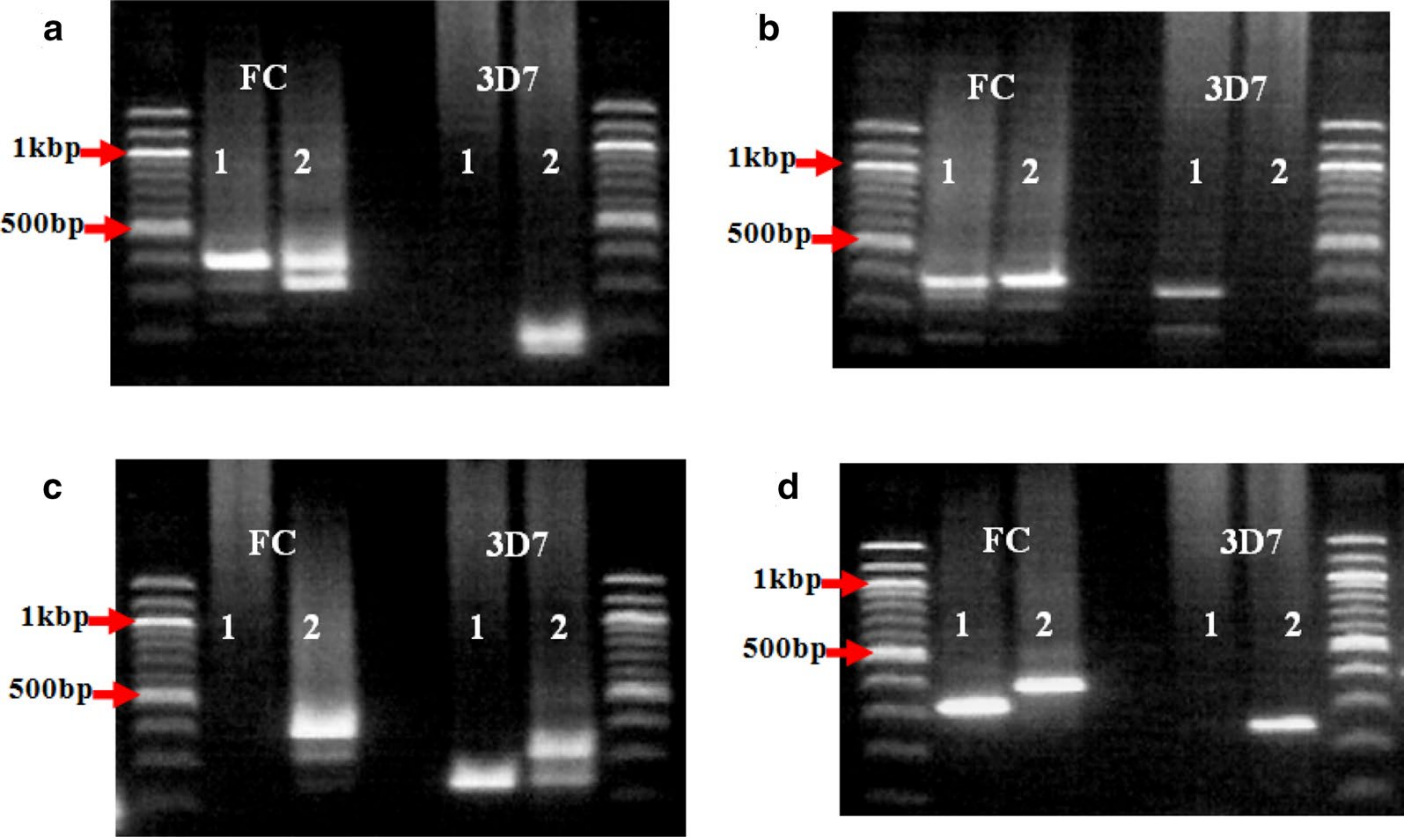
Variations in the distribution of FC27 and 3D7 alleles among children of the same household. **a** A household where child 1 had parasites carrying only FC27 alleles while child 2 had parasites carrying both FC27 and 3D7 alleles. **b** A household where child 1 had parasites carrying both FC27 and 3D7 alleles while child 2 had parasites carrying only FC27. **c** A household where child 1 had parasites carrying monoclonal 3D7 allele while child 2 had parasites carrying both FC27 and 3D7 alleles. **d** A household where child 1 had parasites carrying monoclonal FC27 allele while child 2 had parasites carrying monoclonal FC27 and monoclonal 3D7 alleles. The numeric codes represent the children in the respective household

**Fig. 6:**
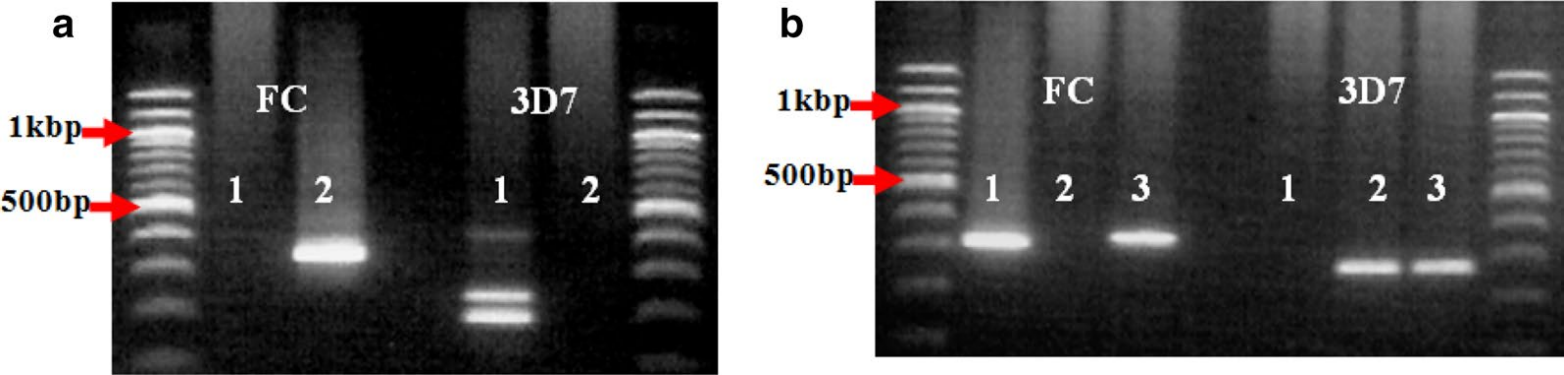
Alleles of the *MSP-2* gene showing unevenly distributed FC27 and 3D7 alleles among children of the same household. **a** A household where the children were infected with only one allele type (either FC27 or 3D7). **b** A household where child 1 had an infection with only FC27 allele type while child 2 had an infection with only 3D7 allele type, but child 3 had mixed infection of both FC27 and 3D7 allele types. In addition, child 1 and 3, as well as child 2 and 3 had an infection with parasites of the same genotypes with respect to their allele types and well as fragment sizes. The numeric codes represent the children in the respective household

In the first household types where both *MSP-2* allele types (FC27 and 3D7) were present, it was observed that in the majority of the households (88.4%, 38/43), both *msp-2* allele were present but disproportionately distributed among the children. In some of these households, all the infected children had both the FC27 and the 3D7 allele types (Fig. 4). In other households (Fig. 5), one of the children had an infection with parasites carrying only one allele type (FC27 or 3D7). In contrast, the other child had an infection with parasites carrying both allelic families (FC27 and 3D7). Furthermore, in some other households within this category, one of the children had parasites carrying only one type of a particular allele (for example FC27allele) while the other child carry the other allele type (in this case, the 3D7 allele) or vice versa (Fig. 6a), but with slight discrepancy (Fig. 6b).

**Fig. 7:**
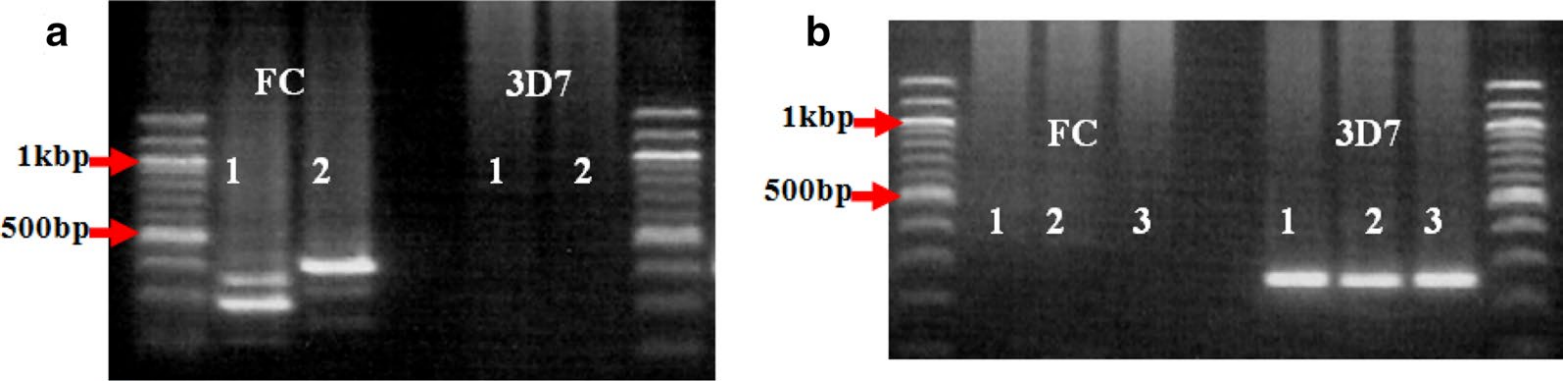
Infection with only one *MSP-2* allele type in all the children of the same household. **a** A household where the children were infected with parasites of the FC27 allelic family only. **b** A household where the children were infected with parasites of the 3D7 allelic family only and all the 3 are of the same genotype with respect to their fragment size. The numeric codes represent the children in the respective household

In the second household types where only one *msp-2* allele (FC27 or 3D7) was present, few households (11.6%, 5/43) have all the children infected with only one type of *MSP-2* allelic family, which may either be the FC27 allele type or the 3D7 allele type (Fig. 7).

## Discussion

The genetic diversity and complexity of *P. falciparum* infections is, to a very large extent, an important indicator of malaria transmission intensity in a region and is a very useful marker for assessing naturally acquired anti-malarial immunity as well as the impact of intervention programmes [9, 35–38]. Numerous studies from different regions have devoted efforts at characterizing the genetic complexity of *P. falciparum* infections at the community level, but very little attention has been given to studying genetic diversity at the level of the micro-environment. In this study, the genetic diversity and complexities of *P. falciparum* infections in the micro-environment was investigated among siblings of the same household. This is the first study to provide information on the genetic diversity and complexity of *P. falciparum* infection at the level of the micro-environment in Nigeria, and it will certainly be a great addition to the limited data available on the subject globally.

The results showed that *P. falciparum* isolates exhibit a remarkable degree of genetic diversity in the micro-environment. Interestingly, it was found that the pattern of distribution of parasite populations within households may be categorized into two based on the prevalence of *msp-2* allelic families. The first category were households where both *msp-2* allele types (FC27 and 3D7) were present, while the second were households where only one *MSP-2* allele type (FC27 or 3D7) was present. The majority of the households (88.4%) investigated belonged to the first category where both *msp-2* alleles were present, showing that parasite clones carrying FC27 and 3D7 alleles are widely distributed in the study region. This observation was in agreement with a previous study in Tanzania where most of the households investigated had parasites of mixed genotypes [39]. An important observation in the households where both *msp-2* allelic families were prevalent was that the FC27 and 3D7 alleles were disproportionately distributed among the infected children. Thus, in some households, all the infected children had mixed allelic infections with parasites carrying both FC27 and 3D7 alleles. In contrast, in other households, one of the children had parasite isolates carrying a particular type of *msp-2* allele and the other child had parasite isolates carrying the other type of *msp-2* allele. Nevertheless, in some other households, one or two of the children may be infected with multiple parasite clones or genotypes which may belong to either of the *msp-2* alleles or both. Although about 65% of the households have at least one child with isolates carrying both the FC27 and the 3D7 allele types, only a few households (30.2%) were observed to have all the children carrying isolates belonging to both the FC27 and the 3D7 allelic families. In a previous study in Gabon, it was observed that about 80% of the members of the household investigated had parasite isolates carrying both FC27 and 3D7 alleles [40] although the study examined only one household. The observed high prevalence of *msp-2* multiclonal infection in this study could be an indication of a high ongoing parasite transmission, suggestive of effective genetic recombination of the parasite population within the female *Anopheles* [41, 42]. Alternatively, it could also have resulted from multiple but independent inoculations of single parasite clones, leading to superinfection. Superinfection is a commonly observed phenomenom in areas of high transmission intensity, especially among individuals with chronic or asymptomatic infections [43], although young children who are yet to acquire immunity, are thought to be protected from superinfection [44].

It was also interesting to note that a few households (11.6%) belonged to the second category, where all the children had parasites carrying only one *msp-2* allele type (FC27 or 3D7). This was also consistent with the findings from Tanzania, where they observed a few instances in which different people in the same household had parasites of similar genotypes [39]. Apart from carrying isolates of the same allele, there were instances also, where identical genotypes or clones (identical fragment sizes) of the same allele were found in all the children in a household. Such infections with parasites of similar genotypes within households might possibly suggest inoculation by a single or related mosquito.

Majority of the participants in this study were infected with a mixture of more than one parasite clone. On the whole, it was found that about 65% of the study participants had polyclonal infections consisting of 2–6 clones with an overall MOI of 2.31 clones per infected child. This observation is consistent with previous reports from Nigeria [10, 31, 42] and from other parts of Africa [14, 24, 45–52]. The simultaneous infection of large number of individuals with multiple parasite genotypes in areas of high transmission intensity has been suggested to be attributable to either multiple inoculations of single clones, or by a single inoculation of multiple clones that may have undergone crossing and recombination in the female *Anopheles* [52, 54]. Recombination events during the sexual stage of the malaria parasites in the *Anopheles* can lead to independent chromosomal re-assortment of genes and is the principal mechanism for generating novel combination of genes, and consequently, new parasite strains with novel genotypes [52–56]. However, diversity may also result from the extensive ectopic recombination events observed during asexual mitotic replication [57]. Nevertheless, there are indications that high genetic diversity in the parasite population might lead to a gradual selection of more virulent strains which in turn can lead to the emergence and proliferation of drug-resistant parasites [10, 58, 59].

The present study has some limitations, including: i) the use of a single genetic marker; and, ii) the fact that PCR may not be able to resolve between alleles of similar size but different sequences or those with a size difference of about 10 bp. All of these may potentially underestimate the complexity of infections in this study. However, these data have provided more insight into the genetic complexity of *P. falciparum* in the micro-environment. Future studies will need to take cognisance of the above limitations, use more robust techniques, and consider other regions with different malaria transmission intensity.

## Conclusion

This study showed high genetic complexity of *P. falciparum* populations in the micro-environment. This is an indication of intense ongoing parasite transmission in the study region and shows that the micro-environment should be a priority target for appropriate malaria control interventions.

## Data Availability

Not applicable
